# Suboptimal Nutrition and Low Physical Activity Are Observed Together with Reduced Plasma *Brain-Derived Neurotrophic Factor* (BDNF) Concentration in Children with Severe Cerebral Palsy (CP)

**DOI:** 10.3390/nu11030620

**Published:** 2019-03-14

**Authors:** Solvejg L. Hansen, Jakob Lorentzen, Lin T. Pedersen, Frederikke L. Hendrich, Martin Jorsal, Jessica Pingel, Jens B. Nielsen, Bente Kiens

**Affiliations:** 1 Molecular Physiology Section, Department of Nutrition, Exercise and Sports, University of Copenhagen, 2100 Copenhagen Ø, Denmark; lintinangonpedersen@gmail.com (L.T.P.); frederikke52@hotmail.com (F.L.H.); bkiens@nexs.ku.dk (B.K.); 2 Department of Neuroscience, University of Copenhagen, 2200 Copenhagen N, Denmark; jlo@elsassfonden.dk (J.L.); jpingel@sund.ku.dk (J.P.); jbnielsen@nexs.ku.dk (J.B.N.); 3 Elsass Institute, 2920 Charlottenlund, Denmark; 4 Geelsgårdskolen, Region Hovedstaden, 2830 Virum, Denmark; martin.jorsal@regionh.dk

**Keywords:** dietary registration, maximal oxygen uptake, blood sampling, energy, dietary fibers, n-3 fatty acids, DHA, plasticity, cerebral palsy

## Abstract

Brain-derived neurotrophic factor (BDNF) is a mediator of exercise and nutrition-induced neural plasticity. In children with cerebral palsy (CP), neuromuscular deficits and mobility impairment have a negative impact on their physical activity level and nutritional status, but whether these children have reduced BDNF concentrations is unknown. Therefore, the aim of the present study was to investigate the plasma BDNF concentration, nutritional status, and physical activity level in children with mild to severe CP. Blood sampling, dietary registration, and questionnaires were completed for children with mild CP (gross motor function classification system (GMFCS) I–II, *n* = 31, age 10.6 ± 0.6 years), severe CP (GMFCS IV–V, *n* = 14, age 10.9 ± 1.1 years) and typically developed (TD) children (*n* = 22, age 10.9 ± 0.6 years). Children with severe CP had ~40% lower plasma BDNF concentration than TD children (*p* < 0.05). Furthermore, children with severe CP had lower daily physical activity level than TD children (*p* < 0.01), and a daily intake of energy, n-3 fatty acids, and dietary fibers that was only ~50% of TD (*p* > 0.001). Reduced plasma BDNF concentrations were observed in children with severe CP. This may be of significance for optimal neural growth and plasticity. This was observed together with low physical activity levels and a suboptimal intake of energy, n-3 fatty acids, and dietary fibers.

## 1. Introduction

There has been an intense focus on daily physical activity level and nutritional status in children the last two decades. This is partly because of strong associations between sedentary behavior, suboptimal nutrition, and childhood obesity [1], but also because physical activity and nutritional status can influence brain plasticity and function as well as learning and memory [2,3]. In children with cerebral palsy, neuromuscular deficit and mobility impairment have a negative impact on these parameters. Cerebral palsy (CP) is an umbrella term, which covers lesions of the developing brain prior to or around the time of birth causing an impaired development of movement abilities and frequently also perceptual and cognitive problems [4]. CP is linked to impaired activity-dependent differentiation and the establishment of connectivity in neural networks during development [5,6,7,8]. Studies have reported lower levels of daily physical activity [9] and physical fitness [10,11,12] in children with CP compared to typically developed (TD) control children. In addition, a high risk of malnutrition due to the insufficient intake of dietary energy has been identified in children with CP in both high [13] and low/middle-income [14,15,16] countries.

The n-3 fatty acid docosahexaenoic acid (DHA) is one of the most promising dietary components in relation to cognitive development in rodents and humans [3]. Studies in rodents have reported that DHA is able to promote synaptogenesis and improve learning and memory abilities [3,17,18,19,20,21]. In addition, DHA has been shown to increase the production of brain-derived neurotrophic factor (BDNF) [19], which is a member of neurotrophic family of proteins, and the most abundant neurotrophic factor in the brain [22]. Neurological development of the brain is highly dependent on BDNF due to its vital role in the growth, survival, and proliferation of distinct neuronal populations in the central nervous system [23,24,25]. In addition, the intake of energy [26,27] and dietary fibers [28,29] has also been shown to influence BDNF production and cognitive development.

In children, studies have shown positive impacts of exercise on cognitive function such as greater attention and working memory [30,31], faster speed at information processing [30], and higher scores in academic achievement tests [32,33] in physically fit children when compared to lower fit peers. BDNF has been suggested to be one of the mediators responsible for the exercise-induced increase in cognitive performance [34]. However, the exact link between BDNF production and improved cognitive performance has not been fully clarified, and is especially difficult to establish in humans, partly because it is not possible to measure brain levels of BDNF. Aerobic exercise and resistance training in adults have been shown to increase serum BDNF concentration in some [35,36], but not all studies [37]. Interestingly, in rodents, DHA supplementation seems to have synergistic effects with exercise/physical activity on the production of BDNF and on learning and memory [3,38,39].

The combination of low physical activity, low energy intake, and malnutrition in children with CP may be accompanied by insufficient production of BDNF and thereby suboptimal conditions for neurological development. However, this remains to be elucidated. The aim of the present study was to determine age–body weight *z*-scores, energy and nutritional intake (including intake of n-3 fatty acids and fibers), daily physical activity level, physical fitness level (when feasible), and circulating concentrations of BDNF and other blood parameters in children with mild and severe CP and a group of TD children.

## 2. Materials and Methods

### 2.1. Subjects

A total of 22 typically developed (TD) control children (13 boys and nine girls, mean age 10.9 ± 0.6 years), 31 children with mild CP (19 boys and 12 girls, mean age 10.6 ± 0.6 years), and 14 children with severe CP (seven boys and seven girls, mean age 10.9 ± 1.1 years) were recruited for the study. Children with CP were recruited in collaboration with the Elsass Institute, 2920 Charlottenlund, Denmark, Geelsgårdskolen, 2830 Virum, Denmark and Department of Orthopedic Surgery, Copenhagen University Hospital Hvidovre, 2650 Hvidovre, Denmark. Children with CP were classified using the gross motor function classification system (GMFCS) [40], with mild CP ranging from level I to II (CP I–II) and severe CP ranging from level IV to V (CP IV–V).

The subjects performed dietary and activity registration (completion rate: TD: 73%, CP I–II: 42%, CP IV–V: *n* = 79%) and blood sampling (completion rate: TD: 68%, CP I–II: *n* = 42%, CP IV–V: 79%). Furthermore, TD and CP I–II performed maximal oxygen uptake testing (completion rate: TD: 50%, CP I–II: 35%).

### 2.2. Ethics Approval and Consent to Participate

The children and their parents gave informed written and oral consent to the study, which was approved by the local ethics committee for the Copenhagen area (H-B-2009-017). The study was performed in accordance with the Helsinki Declaration.

### 2.3. Measurements of Height and Body Weight

A digital physician scale with a height rod was used to measure body weight and height in TD, CP I–II, and CP IV–V, when feasible. In non-ambulatory children, height was measured with a height rod in a supine lying position on an examination bed, and weight was measured with a digital scale weight (Guldmann, Aarhus, Denmark) by lifting the child in a lift sheet. The children were measured while fasting in the morning whenever feasible.

### 2.4. Diet and Activity Registration

Four to eight days of individual dietary registrations, where all food items and beverages were weighed within 1 g of accuracy, were completed to examine the daily habitual diet composition and energy intake. Information and instructions were provided verbally and in writing to the children, a family member, and employees of the child’s school. Representative days for diet recording were planned together with each child and family member. Intake of macronutrients, micronutrients, and energy were calculated using the computer program Vitakost (Vitakost, Kolding, Denmark). The children and their parents also filled out physical activity questionnaires (type, duration, and intensity of daily activities) on the same days as diet registrations were performed. Nordic Nutrition Recommendations 2012 [41] were used as a reference for the recommended intake of energy, macronutrients, and micronutrients.

### 2.5. Maximal Oxygen Uptake

Physical fitness level was evaluated in CP I–II and TD by measurements of maximal oxygen uptake (VO2 max) during an incremental treadmill running test to exhaustion, as previously described [42]. In brief, the protocol started with 1 min of running at an individual maximal running speed of 9–11.5 km/h on a slope of 0% followed by a stepwise 1% incline every 1 min until exhaustion. The criteria for reaching exhaustion were: respiratory exchange ratio (RER) >1.00, a plateau in VO_2_ despite increasing slope, and inability to continue running despite verbal encouragement. During all of the tests, the children were secured with a safety belt (Teddy pants, Liko, Sweden) and breathed through pediatric masks adapted to their faces. Respiratory gas exchanges were measured breath-by-breath using an automatic gas analysis system (CPX MedGraphics, Saint Paul, USA) to determine oxygen uptake (VO_2_) and respiratory exchange ratio (RER). 

### 2.6. Blood Sampling

Children arrived in the morning in a fasting state (12 h) and after, 15 min of rest in a sitting or lying position, blood was drawn from an antecubital vein. Plasma and serum samples were frozen at −30 and −80 °C, respectively, until further analysis. 

Blood samples (*n* = 7) were taken from children with CP undergoing surgery during anesthesia. Blood samples were kept on ice during the surgery and centrifuged at 3000 rpm. Then, plasma was transferred into Eppendorf tubes and stored at −80 °C until subsequent analysis.

### 2.7. Analysis

Plasma glucose concentration was measured using ABL815 (Radiometer Medical A/S, Copenhagen, Denmark). Quantitative determination of insulin in plasma was performed by sandwich-type immunoassay (Triolab, Alpco, New Hampshire, USA). Plasma BDNF concentration was measured by a commercially available kit (Cat. No. CYT306, Chemicon International Chomikine) by ELISA (Millipore, Corporation, Massachusetts, USA). Serum sodium and potassium were determined by flame photometer, FLM3 (Radiometer, Brønshøj, Denmark), and total plasma cholesterol by spectrophotometric analysis. All analyses were performed in accordance with the recommendations of the manufacturer.

### 2.8. Calculations

As recommended by World Health Organization (WHO), *z*-scores must be used as a global reference for the definition of malnutrition. Data from Rigshospitalet (www.vækstkurver.dk) were used as growth standards describing the development of healthy children in optimal conditions in Denmark. Thus, we identified the deviation of growth of Danish children with CP from those of healthy Danish children in optimal conditions. SD-score was calculated as (X/M)^L^ − 1/L*S, if L ≠ 0 with X being the value of interest (height, body weight, and body mass index (BMI)) and L, M, and S being values obtained from the standards depending on age and gender. 

Resting energy expenditure (REE) was estimated in accordance with Henry 2005 [43]. Information from the activity questionnaires was used to estimate a physical activity (PAL) score in accordance with Nordic Nutrition Recommendations 2012 [41]. Daily energy expenditure (EE) was calculated as PAL*REE.

The homeostasis model assessment insulin resistance index (HOMA-IR) was calculated as fasting insulin (µU/mL)*fasting plasma glucose (mmol/L)/22.5 [44].

### 2.9. Statistics

Data are expressed as means ± standard error of the mean (SEM). All data were analyzed using SigmaPlot (version 11.0, SYSTAT Software, San Jose, CA, USA). One-way analysis of variance (ANOVA) tests with Tukey post-hoc test were performed on results comparing all three groups (TD, CP I–II, and CP VI-V) except when testing for group differences in the regression slopes. Here, one-way analysis of covariance (ANCOVA) tests with Tukey post-hoc test were performed. In the treadmill testing, unpaired *t*-tests were performed as results included only two groups (TD and CP I–II). A significance of *P* < 0.05 was chosen in all cases.

## 3. Results

### 3.1. Subject Characteristics 

The subject characteristics of all included children are presented in Table 1. The control group of TD children was comparable to healthy Danish children in relation to age–height, age–body weight, and age–body mass index (BMI) *z*-scores. There was no difference in age between TD, CP I–II, and CP IV–V. Height, body weight, and BMI in average and as *z*-scores did not differ between TD and CP I–II. In contrast, CP IV–V had ~10% lower height (*p* < 0.05) and ~33% lower body weight (*p* < 0.01) than TD. The height and body weight of CP IV–V were also lower than CP I–II (*p* < 0.001). Furthermore, age–height, age–body weight, and age–BMI *z*-scores of CP IV–V were remarkably low and significantly lower than TD (*p* < 0.05) and CP I–II (*p* < 0.05).

**Figure 1:**
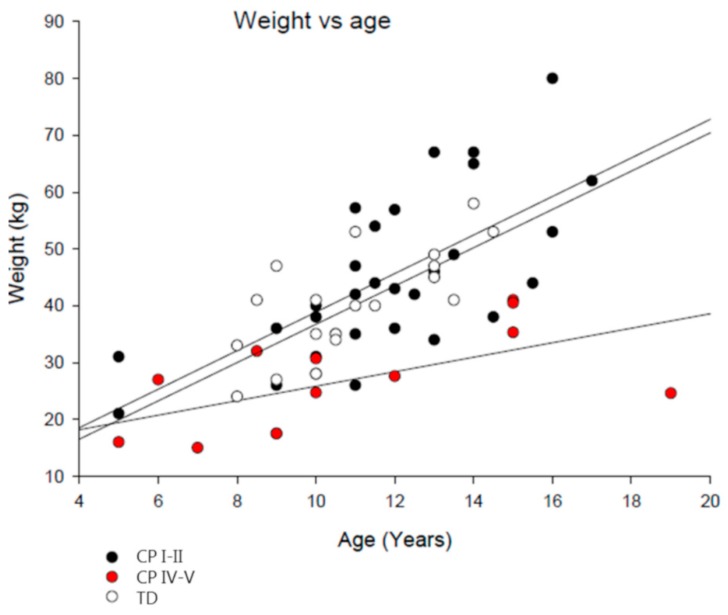
Scatter plot illustrating the relationship between age and body weight in TD children (*n* = 22) and children with CP at GMFCS level I–II (*n* = 31) and IV–V (*n* = 14). A one-way ANCOVA with Tukey post-hoc test was performed.

The relationships between age and weight in the groups are illustrated in Figure 1. When evaluating the effect of age on body weight by one-way ANCOVA with Tukey post-hoc test, the slope of CP IV–V was significantly different from TD (*p* < 0.001) and CP I–II (*p* < 0.001), illustrating a lower increase in body weight with increasing age in CP IV–V than TD and CP I–II. There was no difference in the slope between TD and CP I–II. These data illustrate a deviating relationship between age and body weight in children with severe CP, and thus indicate an abnormal growth rate in this group.

### 3.2. Resting Energy Expenditure (REE) and Daily Energy Expenditure (EE)

Daily physical activity level and body composition were similar in TD and CP I–II. REE averaged to 5.6 ± 0.2 megajoule (MJ)/day in TD and CP II, with no difference between the groups. In contrast, REE in CP IV–V was 4.51 ± 0.2 MJ/day, which was 20% lower than TD and CP I–II (*p* < 0.05). EE was 7.49 ± 0.4 MJ/day in CP IV–V, which was significantly lower by ~30% than TD (9.63 ± 0.3 MJ/day) and CP I–II (9.59 ± 0.3 MJ/day) (*p* < 0.001).

### 3.3. Evaluation of Physical Fitness Capacity 

To investigate physical fitness level, maximal oxygen consumption was measured during running on a treadmill in CP I–II and TD (Table 2). In CP I–II, VO_2_ max averaged 51.4 ± 1.5 mL/min/kg and was not different from TD. Furthermore, there was no group difference in the weekly hours of sport-related activities (Table 2), based on questionnaires, indicating an active CP I–II group. At the end of the treadmill test, the criteria for exhaustion were reached, and the average maximal heart rate and respiratory exchange ratio (RER) were equally high in both groups (Table 2).

### 3.4. Eating Patterns 

**Figure 2:**
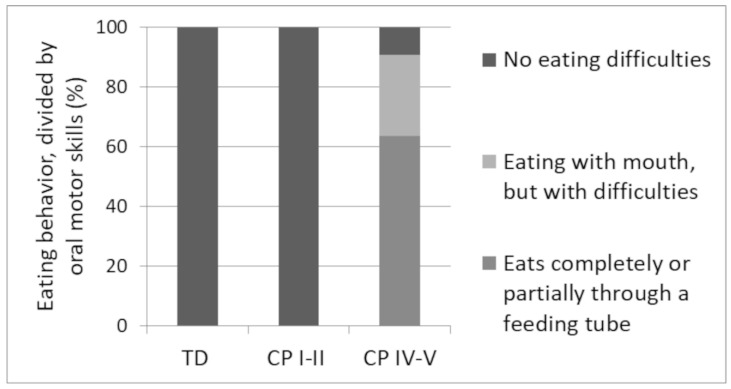
Eating behavior divided by oral motor skills in percentage of TD children (*n* = 16) and children with CP at GMFCS level I–II (*n* = 13) and IV–V (*n* = 11).

In a subgroup of children that performed dietary registrations, different eating behaviors were observed depending on the group (Figure 2). In TD (*n* = 16) and CP I–II (*n* = 13), no eating difficulties were observed. In contrast, normal eating behavior was only observed in ~9% of CP IV–V (*n* = 11). In this group, ~27% of the children were eating orally, but with difficulties, and ~64% of the children were eating partially or completely through a feeding tube (Figure 2).

### 3.5. Daily Intake of Energy and Macronutrients 

**Figure 3:**
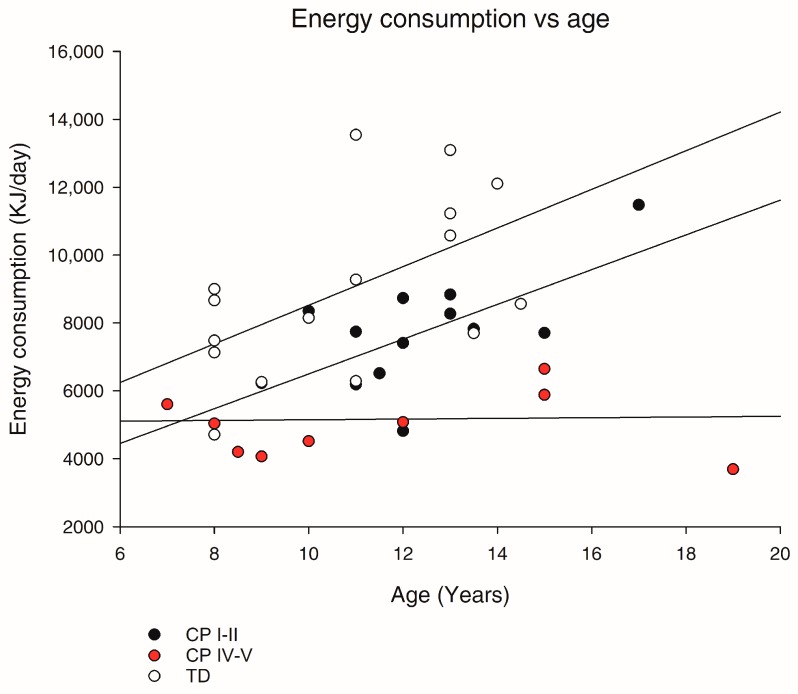
Scatter plot illustrating the relationship between age and energy intake in TD children (*n* = 16) and children with CP at GMFCS level I–II (*n* = 13) and IV–V (*n* = 11). A one-way ANCOVA with Tukey post-hoc test was performed.

The daily energy intake in TD was on average ~9 MJ and within the Nordic Nutrition Recommendations [41] (Table 3). CP I–II did not differ in daily energy intake from TD, whereas CP IV–V had lower daily energy intake than recommended, and ~40% lower daily energy intake than both TD (*p* < 0.001) and CP I–II (*p* < 0.01) (Table 3). When evaluating the relationship between age and energy intake, there was a significantly different slope in CP IV–V compared to TD (*p* < 0.05, Figure 3), illustrating a lower increase in energy intake with increasing age in CP IV–V than TD. 

The intake of macronutrients is listed in Table 3. There were no group differences in protein intake (~16 E%) or daily protein intake expressed as gram per kg of body weight (BW). Dietary fat intake (E%) was ~8 E% higher in CP IV–V than TD (*p* = 0.01). Importantly, daily intake of n-3 fatty acids was ~45% lower in CP IV–V than TD and CP I–II (*p* < 0.001). Four children in CP I–II and two children in CP IV–V supplemented their diet with n-3 fatty acid supplements. Including supplementation, the average intake of n-3 fatty acids was 1.8 ± 0.2 in CP I–II and 0.7 ± 0.1 in CP IV–V. Daily fiber intake was lower in CP IV–V than TD when expressed as E% (*p* < 0.05), in grams (*p* < 0.001), and in grams per MJ energy intake (*p* < 0.05). CP IV–V had lower daily intake of cholesterol than TD and CP I–II (*p* < 0.05) and lower daily intake of added sugar than TD (*p* < 0.001) and CP I–II (*p* < 0.01).

### 3.6. Daily Intake of Vitamins and Minerals 

The intake of vitamins and minerals are listed in Table 4. In TD, the daily intake of the majority of vitamins and minerals were within recommended levels [41], when dietary supplements were excluded. There were no statistical differences in daily intake of vitamins and minerals between TD and CP I–II. Children with CP IV–V had sufficient intake of some vitamins and minerals despite low energy intake. However, the daily intake of vitamin B12, ascorbic acid, beta-carotene, phosphorus, and potassium were significantly lower in CP IV–V than TD. Interestingly, CP IV–V had a significantly higher daily intake of vitamin D than TD and CP I–II (*p* < 0.01). However, without dietary supplementation, the majority of children in all groups did not meet the recommended level of vitamin D intake (TD: ~88%, CP I–II: ~93%, CP IV–V: ~55%). Children who ingested vitamin D supplementation (TD: *n* = 5, CP I–II: *n* = 6, CP IV–V: *n* = 5) met the recommended intake when supplementation was included, and thus, the percentage of children who did not meet the recommended intake of vitamin D was reduced when adding dietary supplementation (TD: ~56%, CP I–II: ~46%, CP IV–V: ~27%).

### 3.7. Fasting Blood Parameters 

**Figure 4:**
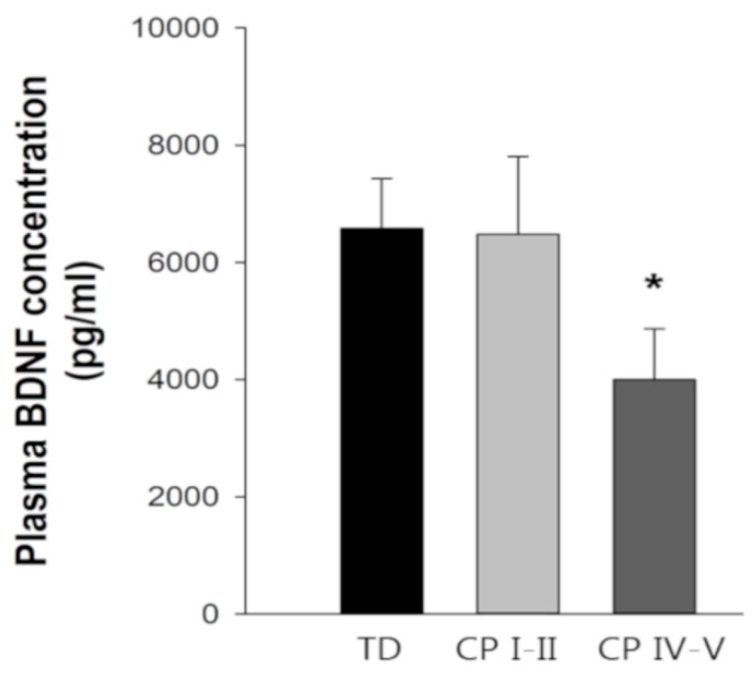
Fasting plasma *brain-derived neurotrophic factor* (BDNF) concentration in TD children (*n* = 15) and children with CP at GMFCS level I–II (*n* = 13) and IV–V (*n* = 11). Data are means ± SEM. One-way ANOVA with Tukey post-hoc test was performed. * *p* < 0.05 compared to TD.

The fasting plasma BDNF concentration averaged ~6500 pg/mL in TD and CP1-II (Figure 4). Notably, in CP IV–V, fasting plasma BDNF concentration was ~4000 pg/mL, which was ~40% lower than in TD (*p* < 0.05). 

Fasting plasma glucose concentration averaged 5 mmol/L in all groups (Table 5). Furthermore, there were no statistically significant differences in fasting insulin concentration or HOMA-IR, but the variations were high, especially in CP IV–V. There was no difference between TD and CP I–II regarding fasting profile. In contrast, CP IV–V had significantly lower concentrations of serum sodium than TD (*p* < 0.05, Table 5).

## 4. Discussion

In the present study, we observed a deviating relationship between age and body weight in children with severe CP compared to children with mild CP and TD children, which was supported by a ~33% lower average body weight in children with severe CP than age-matched TD and age-body weight *Z*-scores in an average −4 of standard scores. In contrast to children with severe CP, we observed normal growth parameters in children with mild CP when compared to TD children and judged by age *Z*-scores. The low body weight in children with severe CP may be related to low energy intake rather than high energy expenditure. Low energy intake in children with severe CP resulted in an insufficient intake of n-3 fatty acids and dietary fibers. Interestingly, in children with severe CP, but not mild CP, plasma BDNF concentration was ~40% lower than in TD children. This could be of significance for optimal neural growth and plasticity.

In the included children with severe CP, eating difficulties were highly represented, with more than 60% of the children eating completely or partially by feeding tube, whereas no eating difficulties were observed in children with mild CP or TD children. The eating difficulties could explain the observed low energy intake in the group with severe CP. Although the dietary record method has some limitations, such as for example risks of underreporting, the energy intake in the children with severe CP was 40% lower than TD children. Furthermore, low energy intake in children with severe CP is a notion that is supported by others [13,45]. The present data, together with the existing knowledge linking low energy intake to poor linear growth in children with CP [46,47,48,49,50], indicate that the low energy intake with age underlies the non-linear growth in children with severe CP in the present study.

A high risk of inadequate energy for growth in children with severe CP is a concern even though the present group of severe CP had significantly lower resting and daily energy expenditure than children with mild CP and TD children. However, it should be noted that daily energy expenditure was estimated based on activity questionnaires and body weight and height, which has some limitations. Although the resting energy expenditure and daily energy expenditure of the TD group was not different from previous reports in children within a similar age range (review in [51]), it can be speculated that increased muscle activity, due to the involuntary movements in children with severe CP, may increase resting energy expenditure and energy expenditure in different positions, and thus makes the low energy intake even more detrimental. In fact, it was recently reported that children with moderate CP have 40% higher energy expenditure during standing than their TD peers, which was measured as oxygen consumption [52]. Furthermore, in a study by Verschuren et al. (2014), energy expenditure during different positions was measured by indirect calorimetry in children with a variety of CP classifications. Despite the small sample sizes, the authors were able to detect increased energy expenditure during standing with increasing disability [53]. In line with this, children with CP have a higher energy [11] and oxygen [54] cost for walking compared to TD, and the oxygen cost increases with increasing disability [55]. Collectively, these data indicate elevated energy expenditure during different positions in children with severe CP and the present data on energy expenditure, in this group, may likely be underestimated.

Another consequence of the low energy intake in children with severe CP was the low intake of n-3 fatty acids, which was ~45% lower than the TD group. The n-3 fatty acid DHA has been shown to be an important component of the cell membrane of neurons of the central nervous system, where it may provide fluidity of the membrane at synaptic regions [56]. This may facilitate synaptic plasticity through mechanisms such as increased integration and the movement of receptors and other membrane proteins that are essential for establishing new synapses [17,57]. DHA has also been shown to increase the production of BDNF, which is one of the neurotrophic factors involved in synaptic plasticity [19,38,58]. It is probably these mechanisms which are responsible for the positive effect of DHA supplementation on learning and memory, which has been documented in rodents [38,59,60]. Human studies have shown mixed results [42,61,62,63,64]. The low intake of n-3 fatty acids in children with severe CP was observed despite n-3 fatty acid supplements in many of the feeding tube products.

In the present study, low dietary fiber intake was observed in children with severe CP compared to TD children. A recent study reported increased plasma BDNF concentration as a result of fiber-rich dietary intake in healthy adults [28]. Furthermore, in healthy children, dietary fiber intake has been positively related to nutrient energy intake [65], and attentional inhibition as resisting distractions and habits to maintain focus [29], suggesting an important role of dietary fibers in children with CP. In addition, in this group of children with severe CP, the physical activity level was very low. BDNF has been suggested to be one of the mediators responsible for the exercise-induced increase in cognitive performance [34], and DHA supplementation has been shown to act synergistically with exercise/physical activity on the production of BDNF and on the learning and memory abilities of rodents [3,38,39]. On the basis of these observations, it can be speculated whether the reduction in n-3 fatty acid and dietary fiber intake per se and/or combined with low physical activity level contribute to the 40% lower plasma BDNF concentration in this group of children with severe CP compared to TD children. Supporting this is the adequate intake of n-3 fatty acids, dietary fiber, and daily physical activity level, together with similar high plasma BDNF concentrations in children with mild CP and TD children. In the literature, there is currently only limited data on circulating BDNF concentration in children with CP. In one study, three-year-old children with moderate to severe CP did not differ in neonatal blood BDNF concentration from TD children [66]. However, this study did not account for nutrient status. In order to unravel the effects of physical activity and different nutrients on plasma BDNF concentrations in TD children, and especially in children with CP, more studies are needed.

BDNF can cross the blood–brain barrier, and blood drawn from the antecubital vein is believed to reflect the BDNF levels in the brain [67]. However, although the major production of BDNF is in the brain, peripheral tissue such as skeletal muscle [68] and brown and white adipose tissue [69] can also produce BDNF and may add to the concentration of circulating BDNF. Although no direct measurements of muscle mass or fat mass were performed in the present study, the markedly low body weight in children with severe CP can presumably partly be ascribed to a lower muscle mass and fat mass, as reported by others [70]. This may contribute to the low circulating BDNF concentration in this group. One possible consequence of low plasma BDNF concentration in children with severe CP is an increased risk of insufficient brain BDNF concentration, which may be of significance for optimal neural growth and plasticity. Thus, the observational data from the present study suggest optimizing BNDF-facilitating nutrients and physical activity to increase the conditions for brain development.

The suboptimal nutrition that characterized children with severe CP, in terms of low energy intake and insufficient intake of n-3 fatty acids and dietary fibers, identifies a challenge for caregivers and parents and nutritional counseling, and feeding strategies are necessary. One strategy to increase energy intake is by increasing the use of tube feeding. In a study by Sullivan et al. 2005, feeding by gastrostomy in children with CP resulted in increased energy intake and body weight [71]. That tube feeding is superior to oral feeding in terms of increasing body weight is supported by comparative studies on children with CP, reporting a higher incidence of children with CP who were low in age-related weight when orally fed compared to gastronomy tube fed [72,73]. However, the orally fed children, in one of these studies, had a higher intake of dietary fibers than the gastronomy tube children [72]. This highlights the importance of choosing the right tube-feeding product.

The low intake of n-3 fatty acids in children with severe CP identifies a need for an increased intake of n3-rich products or dietary supplementations such as DHA supplementation. DHA supplementation has previously been shown to increase circulating DHA in healthy preschool children [42], and might therefore be one recommended solution in order to ensure sufficient levels in children with severe CP.

Low dietary fiber intake in children has been reported as a risk factor for chronic constipation [74], which is a comorbidity that is common in CP [75,76]. The normalization of dietary fiber intake is recommended as a first strategy for treating constipation in children with CP [75]. Thus, in children suffering from constipation, it is important to ensure sufficient dietary fibers to the diet and tube products to improve the function of the gastrointestinal system.

In contrast to children with severe CP, children with mild CP displayed a similar nutritional intake as their TD peers in terms of comparable levels of energy intake and dietary intake of micronutrients and macronutrients. One explanation may be normal feeding behavior without any feeding difficulties. Another explanation could be that this group had comparable body composition, daily activity levels, and weekly sport activities as the TD group. In order to investigate whether this normal activity level of children with mild CP also translated to normal physical fitness, we measured VO_2_-max by treadmill testing to exhaustion in children with mild CP and TD children. VO_2_-max is considered a good marker of physical fitness status, and school-based programs have illustrated that increased daily physical activity can increase VO_2_-max in children and adolescents [77,78,79]. VO_2_-max in the control group was on average 52 min/min/kg, which is comparable to [80,81] or higher than [82,83] other studies on adolescent or typically developed children, but lower than athletic children [80]. Interestingly, children with mild CP did not differ in VO_2_-max compared to their TD peers in the present study, which is in contrast to other studies reporting a lower VO_2_-max in children with CP than healthy peers [10,11,12]. Differences in the level of daily physical activity could explain these differences. No measure of daily physically activity level was reported in two of the studies [10,11], and more children in the control group than in the CP group classified themselves as physically “active” and “very active” in the third study [12]. Also, when comparing the absolute VO_2_-max values with the CP groups across these studies, VO_2_-max in the present CP group (51.4 ± 1.5) was markedly higher than previously reported (28.7–42 mL/min/kg [10,11,12]), supporting an active CP group in the present study. These data indicate that active children with mild CP have a similar physical fitness status to TD children, which highlights the importance of encouraging children with CP to be physically active.

## 5. Conclusions

A significantly reduced plasma BDNF concentration was observed in children with low body weight and severe CP. This was observed together with low physical activity level and a suboptimal intake of energy, n-3 fatty acids, and dietary fibers. The suboptimal dietary intake might be due to eating difficulties. In contrast to children with severe CP, body weight, eating behavior, dietary intake, physical activity level, and physical fitness status was normal in children with mild CP, and fasting plasma BDNF concentration was similar to their TD peers. Together, these data suggest that individually-based dietary counseling should be recommended for children with severe CP, with a focus on n-3 fatty acids supplementation and the sufficient intake of energy and dietary fibers, as such actions may optimize neural growth and plasticity.

## Figures and Tables

**Table 1:** Subject characteristics.

	TD	CP I–II	CP IV–V
Number of children	22	31	14
Boys/girls	13/9	19/12	7/7
Age (years)	10.9 ± 0.6	10.6 ± 0.6	10.9 ± 1.0
Height (cm)	147 ± 3	152 ± 3	131 ± 5 *^,###^
Age–Height (z-score)	0.1 ± 0.2	0.0 ± 0.2	−2.4 ± 0.7 ***^,##^
Body weight (kg)	40 ± 2	45 ± 3	27 ± 2 **^,###^
Age–Body weight (z-score)	0.2 ± 0.2	0.2 ± 0.3	−4.0 ± 1.2 **^,###^
BMI (kg/m^2^)	18 ± 1	19 ± 0.6	16 ± 1 ^(^*^),##^
Age–BMI (z-score)	0.1 ± 0.3	0.3 ± 0.2	−1.2 ± 0.5 *^,##^

Data are obtained from typically developed (TD) children and children with cerebral palsy (CP) at gross motor function classification system (GMFCS) level I–II and IV–V. Data are means ± SEM. One-way ANOVA with Tukey post=hoc test was performed. ^(^*^)^ *p* = 0.1, * *p* < 0.05, ** *p* < 0.01 *** *p* < 0.001 vs. TD, ^##^ *p* < 0.05, ^###^ *p* < 0.001 vs. CP I–II. BMI: body mass index.

**Table 2:** Physical fitness capacity determined as maximal oxygen uptake (VO_2_ max).

	TD	CP I–II
Sport-related activities (hours per week)	2.5 ± 0.8	2.3 ± 0.5
Heart rate max	197 ± 3	197 ± 3
RER	1.2 ± 0.02	1.1 ± 0.02
VO_2_ max (L/min)	2.0 ± 0.2	2.2 ± 0.1
VO_2_ max (mL/min/kg)	52.5 ± 2.5	51.4 ± 1.5

Testing was performed in TD children (*n* = 11) and children with CP at GMFCS level I–II (*n* = 11). Data are means ± SEM. RER: respiratory exchange ratio.

**Table 3:** Daily intake of energy and macronutrients.

	TD	CP I–II	CP IV–V	RI *^a^*
Energy Intake (KJ)	8983 ± 633	8144 ± 540	5165 ± 291 ***^,##^	Girls: 8600 Boys: 9300
**Macronutrients**				
Protein (E%)	16.7 ± 0.6	15.4 ± 0.6	15.3 ± 1.4	10–20
Protein (g/kg BW)	2.22 ± 0.14	1.81 ± 0.14	1.77 ± 0.53	0.75
Carbohydrates (E%)	52.5 ± 1.4	50.3 ± 0.9	46.9 ± 1.9 *	45–60
Dietary fibers (E%)	2.6 ± 0.2	2.4 ± 0.1	1.5 ± 0.5 *	
Dietary fibers (g)	28.5 ± 1.8	23.9 ± 2.0	9.6 ± 2.9 ***^,#^	>10
Dietary fibers (g/MJ)	3.3 ± 0.2	2.9 ± 0.1	2.0 ± 0.4 *	2–3
Fat (E%)	28.3 ± 1.4	32.0 ± 1.0	36.2 ± 10.9 *	25–40
Saturated fatty acid (E%)	10.1 ± 0.7	10.5 ± 2.5	11.1 ± 3.4	<10
Monounsaturated fatty acid (E%)	8.0 ± 0.5	8.9 ± 0.5	12.0 ± 3.8	10→20
Polyunsaturated fatty acid (E%)	3.7 ± 0.2	4.6 ± 0.4	5.1 ± 1.6	5→10
n-3 fatty acids (g)	1.5 ± 0.2	1.6 ± 0.2	0.6 ± 0.1 ***	
**Cholesterol (mg)**	198 ± 22	213 ± 22	103 ± 26 *^,#^	<300
**Added sugar (E%)**	8.9 ± 1.3	8.8 ± 1.8	4.9 ± 3.9 ***^,##^	<10

Data are given by an average of three to eight independent days of 24-h dietary weighing in TD children (*n* = 16) and children with CP at GMFCS level I–II (*n* = 13) and IV–V (*n* = 11). Data are means ± SEM. One-way ANOVA with Tukey post-hoc test was performed. * *p* < 0.05, *** *p* < 0.001 vs. TD ^#^ *p* < 0.05, ^##^ *p* < 0.01 vs. CP I–II. BW: body weight. E%: percentages of total energy intake. *^a^* RI: Recommended intake for a child aged 10–11 years from Nordic Nutrition Recommendations (NNR) 2012 [41].

**Table 4:** Daily intake of micronutrients.

	TD (*n* = 16)		CP I–II (*n* = 13)		CP IV–V (*n* = 11)		RI *^a^*
	Dietary Intake	Incl. Dietary Supplements	Dietary Intake	Incl. Dietary Supplements	Dietary Intake	Incl. Dietary Supplements	
**Vitamins**							
Vitamin A (RE (µg))	945 ± 124	1045 ± 130	737 ± 146	890 ± 167	865 ± 214	1083 ± 205	600
Vitamin D (µg)	3.3 ± 0.9	6.4 ± 1.3	3.0 ± 1.0	11.3 ± 4.4	8.7 ± 1.7 **^,##^	20.0 ± 5.6	10
Vitamin E (aTE (mg))	6.5 ± 0.6	7.6 ± 1.1	8.0 ± 1.1	10.7 ± 2.0	8.8 ± 1.7	11.4 ± 1.6	7
Vitamin B1 (thiamin) (mg)	1.4 ± 0.1	1.6 ± 0.1	1.2 ± 0.1	1.5 ± 0.2	1.2 ± 0.2	1.5 ± 0.2	1.2
Vitamin B2 (riboflavin) (mg)	1.5 ± 0.1	1.7 ± 0.1	1.4 ± 0.2	1.8 ± 0.3	1.2 ± 0.2	1.6 ± 0.2	1.4
Vitamin B3 (niacin) (mg)	15.0 ± 1.8	17.9 ± 1.8	13.0 ± 2.3	17.5 ± 2.4	12.1 ± 1.8	16.6 ± 2.0	Girls: 14, Boys: 16
Vitamin B6 (mg)	1.6 ± 0.2	1.9 ± 0.2	1.4 ± 0.1	1.8 ± 0.2	1.4 ± 0.2	1.8 ± 0.2	1.3
Vitamin B12 (µg)	4.4 ± 0.4	9.2 ± 1.5	4.5 ± 0.6	5.2 ± 0.7	2.5 ± 0.4 **^,##^	3.1 ± 0.8	2
Vitamin C (mg)	120 ± 17	137 ± 15	115 ± 8	143 ± 18	94 ± 12	116 ± 13	50
Ascorbic acid (mg)	70 ± 3		70 ± 9		27 ± 8 *^,##^		
β-carotene (µg)	7241 ± 1391		3707 ± 790		1879 ± 698 **		
**Minerals**							
Calcium (mg)	960 ± 88	979 ± 82	794 ± 117	842 ± 117	751 ± 102	987 ± 113	900
Phosphorus (mg)	1359 ± 86	1359 ± 85	1163 ± 116	1174 ± 114	675 ± 60 ***^,##^	675 ± 59	700
Potassium (mg)	2827 ± 224		2503 ± 212		1294 ± 163 ***^,#^		Girls: 2900, Boys: 3300
Magnesium (mg)	328 ± 24	335 ± 24	272 ± 25	295 ± 33	187 ± 17	190 ± 16	280
Iron (mg)	9.5 ± 0.7	11.2 ± 0.9	7.9 ± 0.5	11.9 ± 1.9	10.1 ± 1.7	14.1 ± 1.7	11
Zinc (mg)	10.7 ± 0.9	11.3 ± 0.8	8.6 ± 0.8	10.7 ± 1.2	8.6 ± 1.4	11.3 ± 1.3	11
Copper (mg)	2.7 ± 0.4	2.8 ± 0.4	2.1 ± 0.3	2.3 ± 0.4	2.8 ± 0.5	3.0 ± 0.5	0.7
Iodine (µg)	137 ± 13	155 ± 12	138 ± 22	178 ± 29	108 ± 14	148 ± 21	150
Selenium (µg)	46 ± 5	51 ± 5	38 ± 4	52 ± 7	40 ± 7	54 ± 7	40

Data are means of three to eight independent days of 24-h dietary weighing in TD children (*n* = 16) and children with CP at GMFCS level I–II (*n* = 13) and IV–V (*n* = 11). Dietary supplementation was ingested in five TD, seven CP I–II, and five CP IV–V. Data are means ± SEM. One-way ANOVA with Tukey post-hoc test was performed between dietary intake without supplementation. * *p* < 0.05, ** *p* < 0.01, *** *p* < 0.001 vs. TD. ^#^ *p* < 0.05, ^##^ *p* < 0.01 vs. CP I–II. RI *^a^*: Recommended intake for a child aged 10–11 years from NNR 2012 [41].

**Table 5:** Fasting blood values.

	TD	CP I–II	CP IV–V
Plasma glucose (mmol/L)	4.9 ± 0.1	5.0 ± 0.2	5.0 ± 0.1
Plasma insulin (µlU/mL)	5.8 ± 0.8	7.5 ± 1.0	9.0 ± 3.0
HOMA-IR	1.3 ± 0.2	1.9 ± 0.1	2.01 ± 0.7
Plasma hematocrit (%)	43.8 ± 1.0	43.3 ± 1.2	42.2 ± 1.4
Plasma hemoglobin (mmol/L)	8.9 ± 0.2	8.8 ± 0.3	8.5 ± 0.3
Serum sodium (mmol/L)	133 ± 1.0	130 ± 1	125 ± 2 *
Serum potassium (mmol/L)	3.88 ± 0.12	4.05 ± 0.08	4.34 ± 0.26
Plasma total Cholesterol (mmol/L)	3.90 ± 0.18	3.64 ± 0.18	3.57 ± 0.17

Blood sampling is performed in TD children (*n* = 15) and children with CP at GMFCS level I–II (*n* = 13) and IV–V (*n* = 11). Data are means ± SEM. One-way ANOVA with Tukey post-hoc test was performed. * *p* < 0.05 vs. TD. BMI: body mass index. BDNF: Brain-derived neurotrophic factor. HOMA-IR: Homeostasis model assessment insulin resistance index.
